# The road to green development: How can carbon emission trading pilot policy contribute to carbon peak attainment and neutrality? Evidence from China

**DOI:** 10.3389/fpsyg.2022.962084

**Published:** 2022-08-24

**Authors:** Junshi Lan, Wenli Li, Xinwu Zhu

**Affiliations:** ^1^School of Marxism, Xinjiang University, Ürümqi, China; ^2^School of Politics and Public Administration, Xinjiang University, Ürümqi, China

**Keywords:** carbon emissions trading, industrial structure upgrading, heterogeneity, green development, carbon peak

## Abstract

Carbon emission trading is not only a market-based instrument but also one of the government’s macro-policies, which is extremely crucial to fulfilling both carbon peak attainment and carbon neutrality goals. For this purpose, this paper adopts a 30-region dataset for the period from 2008 to 2020 in China and employs the difference-in-difference (DID) method to quantify the effect of the carbon emission trading pilot policy (CETP) on carbon emissions on the basis of introducing industrial structure upgrading and green technology innovation as moderating variables. The results show that (1) CETP has a statistically significant dampening effect on carbon emissions, while its carbon emission reduction effect follows a significant strengthening trend as the policy year of CETP implementation is delayed. (2) CETP has a significant carbon emission reduction effect. However, its effect demonstrates a gradual decrease from the eastern to the central and finally to the western regions. (3) CETP can inhibit carbon emissions depending on industrial structure upgrading to a certain extent, and this dependence is significant in the national and eastern regions but not in the central and western regions. (4) CETP’s carbon emission reduction effect is dependent on green technology innovation, which is only revealed in the western region and performs as a dampening effect in the national, eastern, and central regions, but not significantly.

## Introduction

The second industrial revolution was driven by energy consumption around the world’s economic development, while the third industrial revolution also with technological innovation breakthroughs to enhance energy utilization (Huang et al., 2022). Nevertheless, the long-term sloppy development model which sacrifices the environment has contributed to the global climate problem, which constrains the economic development of all nations nowadays, and especially has a significant impact on the living environment of humankind, threatening its prosperity and survival (Yang et al., 2021a; Lingyan et al., 2022). Because of the cross-regional nature of carbon emissions, high carbon emissions have emerged as a shared dilemma in the world (Sharif et al., 2019; Rana et al., 2021). How to fulfill the “green transition and cultivation of low-carbon economy” has been a worldwide consensus (Wang et al., 2022). With 11.9 billion tons of carbon emissions by 2021, China captures 33% of the world’s carbon emissions^1^. To proactively combat against global climate crisis, President Xi Jinping stated in September 2020 that “China intends to intensify its independent national contribution, implement more effective policies and measures, strive to peak carbon peak by 2030, work towards achieving carbon neutrality by 2060” (Hao et al., 2021).

To fulfill both economic development and carbon reduction double objectives under the resource constraint, the Chinese government initially strictly regulates the carbon emissions of each region through government intervention (Yang and Luo, 2020). However, unilateral intervention by the “visible hand” alone easily turns carbon emissions into a “public good.”^2^ Enterprises will try to emit as much carbon as possible in exchange for high economic benefits, theft of emissions is common, and rent-seeking behavior will inevitably occur. Therefore, it has been the direction that China must be considering to seek market-based instruments to tackle this issue (Razzaq et al., 2021). Carbon emission trading (carbon emission trading scheme (ETS)) is an instrument based on market mechanism operation, which is an efficient mechanism to control carbon emissions in a market environment at a low cost (Zhang, 2012). The carbon emission trading scheme first emerged in developed countries. Dalesj, an American, (1968) gave a more comprehensive account of the implementation of emission trading in his book “Pollution, Price of Wealth.” Since 2005, the European Union member states have implemented a carbon emission trading scheme. The scheme sets emission allowances and allocates emission allowances to member states, the sum of which does not exceed the emissions committed under the Protocol.

In contrast, the allocation of emission allowances takes into account historical emissions, projected emissions, and emission standards of member states (Convery, 2009). To fulfill emission reduction targets and compensate for market failures, the Chinese government announced 2011 the establishment of seven pilot areas and the official opening of the national carbon trading market in 2021. Compared with the government’s approach of administrative penalties, the carbon trading policy uses economic incentives to increase the motivation of carbon emission subjects in carbon emission reduction. Carbon emission trading uses market mechanisms to force enterprises to have potential advantages in technological innovation, reducing energy consumption, energy-saving technologies, and realizing environmental and economic dividends. In addition, using market mechanisms to intervene in carbon emissions can help the Chinese achieve the goal of “carbon peak and carbon neutrality.” Therefore, studying and bringing into play the carbon emission reduction effect of CETP are conducive to building a scientific and perfect policy system and promoting economic “green recovery” and high-quality development (Zhang, 2015).

Recently, while promoting a green economy, the Chinese government has engaged in two core areas of industrial structure upgrading and technological innovation to optimize economic structure (Lin and Ma, 2022). Compared to developed countries, there are deficiencies between technological innovation and resource utilization efficiency. China has basically realized a development model as one of the core elements of the supply-side structural reform. By 2021, the output value of China’s three industries will be 7.3, 39.4, and 53.3%, respectively, and the upgrading of the industrial structure plays the role of “pollutant control body” in economic development^3^. Energy conservation and emission reduction rely on green technology innovation and need the power of the market system to support it. To better achieve green transformation, China has proposed to improve green technology innovation based on seeking economic development, resource conservation, pollution reduction, and environmental protection around carbon peaking and carbon neutrality. With the gradual introduction of market mechanisms into environmental policies, it is increasingly important to exert emission reduction effects based on market instruments (Zhang et al., 2020b).

Carbon trading schemes are devised to guide enterprises to reduce carbon emissions at a lower cost and promote green technology innovation to achieve optimal resource allocation and reduce carbon emissions (Narassimhan et al., 2018; Razzaq et al., 2022). Thus, integrating green technology innovation and industrial structure upgrading into CETP’s influence on carbon emissions is scientifically significant, which can provide a decision reference for the government to formulate and improve green economic development policies. So, can the CETP reduce carbon emissions? Under industrial structure upgrading and green technology innovation perspectives, how does the CETP affect carbon emissions? Also, is there any heterogeneity in the impact of pilot emission trading policy on carbon emissions under different geographical locations? The answers and solutions to the above issues are of great theoretical value and practical significance for China to achieve high-quality economic development and provide experience lessons for economies with similar development to China.

This paper seeks to develop the existing research in the following dimensions. First, this paper integrates CETP and carbon emissions into the unified investigation framework to fill in the relevant studies between the two. Second, this paper further scrutinizes the relationship between CETP and carbon emissions from industrial structure upgrading and green technology innovation to obtain novel research findings. Finally, given the regional heterogeneity, this paper divides the research sample into eastern, central, and western regions to quantify CETP’s regional heterogeneity and empirically inform the government on how to formulate differentiated policies.

The remainder of the paper is structured as follows. Section “Literature review” gives the literature review. Section “Materials and methods” provides the methodology, variable definitions, and data sources. Section “Results and discussions” analyzes and discusses the empirical results in detail. Finally, the paper presents the research conclusion, policy implications, deficiencies, and future research directions.

## Literature review

Since recognizing the impressive consequences of carbon emissions on the economy and the environment, scholars have investigated the factors affecting carbon emissions. From the micro-extension to the macro-level, Chen et al. (2010) find that in a household, electricity and fuel consumption and material consumption increase household carbon emissions, while there is a significant correlation between urban population and economic development and urban household carbon emissions. Huang and Wang (2013) develop a ridge regression multiple linear models and find that a 1% increase in population increases carbon emissions by 0.963%, 0.059%, and 0.266% for GDP per capita, energy intensity, and urbanization, respectively, while each 1% increase in the share of tertiary sector leads to 0.093% carbon emission reduction. Di et al. (2013) discovered that the negative contribution of energy structure is smaller. Song et al. (2014) verify that the cumulative effects of energy mix are positive, and the cumulative effect of industrial structure is negative. Fan et al. (2015) argue that the petrochemical industry consumes much energy and has become a major source of carbon emissions. While the economic growth effect is the main driver influencing carbon emissions from the petrochemical industry, the industrial structure effect suppresses carbon emissions. Wang and Yang (2016) analyze that the growing gap between urban and rural direct and indirect emissions, the growth of GDP per capita and population, and changes in intermediate demand and sectoral emission intensity lead to the growth of urban and rural indirect emissions, while decreasing energy intensity, residential consumption rate, urban and rural consumption rates, and consumption structure effects can play a role in carbon emission reduction.

Moreover, taking Tunisia as a research subject, Daldoul (2018) confirms that the transport sector significantly contributes to carbon emissions, while energy efficiency growth in the transport sector leads to a decrease in carbon emissions. Wang et al. (2019) analyze that the share of coal consumption promotes carbon emission scale and carbon emission intensity, and research and development (R&D) intensity and energy efficiency inhibit factors. Zhang et al. (2021) suggest that the value-added of the logistics industry contributes 65.45% to carbon emissions. China and India are the world’s largest coal consumers and the most populous countries. Ahmed et al. (2022) employ the long- and short-term memory (LSTM) method to find that energy consumption has the strongest role on carbon emissions and renewable energy has the weakest role on carbon dioxide emissions in both countries. Liu and Hao (2022) consider scale efficiency as the main constraint affecting carbon efficiency enhancement in the Yangtze River Economic Zone. Yang et al. (2022b) argue that there are many implied carbon emissions in the export trade, while the change in export scale is the major factor affecting the implied carbon export trade.

Following the introduction and implementation of carbon trading, scholars have conducted numerous studies on this market mechanism’s socioeconomic and carbon impacts. Bosello and Roson (2002) argues that the distributional impact of emission trading stems from the difference between two social welfare functions: one that is implicitly maximized in competitive market equilibrium and the other that is implicitly adopted in the choice of a given equity principle. Tang and Song (2013) find that government quotas, market transactions, and purification treatment can be used to balance carbon credits and, in turn, find an optimal production strategy based on carbon emissions. Wu et al. (2014), with a Shanghai pilot, argued that allowance allocation principles should be adjusted in the pilot region to promote changes in the domestic energy mix and improve emissions data disclosure to ensure information symmetry. Liu et al. (2015) argue that carbon trading, as a market mechanism, is an essential tool for mitigating climate change, inaccurate allocation of allowances, imperfect trading mechanisms, and lagging legislation. Dai et al. (2018) evaluate emission trading schemes (ETS) and renewable energy policies, confirming that emission trading is a cost-effective method to help fulfill reduction targets at a lower cost. Zhang et al. (2019) suggest that ETS significantly curbs carbon emission intensity only in Beijing and Guangdong, with insignificant effects in Tianjin, Shanghai, Chongqing, and Hubei. Wang et al. (2021) used a quasi-natural experiment to confirm that emission reduction policies have dynamic cumulative effects and also analyzed that ETS policies promote carbon emission reduction by improving technological innovation. Li et al. (2022) report that both trade and monetary policy uncertainty positively affect carbon price, while the uncertainty of exchange rate policy negatively affects the market price of carbon emission trading. Ma (2022) reveals that CETP significantly lowers the export product quality of non-state enterprises and significantly deteriorates the export product quality of enterprises in high-energy-consuming industries.

In addition to the effect of CETP on carbon emissions, it also impacts green technology innovation. For example, Chen et al. (2021) found that the pilot policy of CETP reduced the share of green patents by about 9.26%, but there was a lag effect. Zhang and Fu (2022) suggests that the price of carbon trading increases the price of green technology innovation. In comparison, green technological innovation in the study of the impact of CETP on carbon emissions, Zeng et al. (2019), Santra (2017), and Desheng et al. (2021) found that green innovation is an effective means to achieve emission reduction. Moreover, carbon emission trading can also have an impact on industrial structure. Li et al. (2020) argue that CETP promotes carbon emission reduction alliance formation, and inter-firm cooperation under this alliance can promote the heightening of industrial structure. Lu (2020) points out that low-carbon alliances contribute to industrial structure upgrading and form a dynamic cycle mechanism. However, some scholars hold different views. For example, Tian et al. (2014) argued that regional differences in industrial structure seriously affect regional carbon emissions. Zheng et al. (2019) believed that industrial structure upgrading suppressed carbon emissions in most regions. Furthermore, Zhang et al. (2020a) show that industrial structure upgrading indirectly increases carbon intensity by promoting technological change.

Judging from the literature sorting, there is still a gap in the research field about carbon emission trading, and the existing empirical evidence of China’s carbon market is limited. Most current studies have used quasi-natural experiments to test the impact of CETP on carbon emissions, but the results are very different and have not yet reached a consensus. Green technology innovation and industrial structure upgrading are an important direction for carbon trading to force socioeconomic development with economic benefits and a direct impact on carbon emissions. However, the literature that introduces green technology innovation and industrial structure upgrading to CETP affecting carbon emissions is scarce. This paper empirically tests the nexus between CETP and carbon emissions by using a difference-in-difference model (DID). In addition, this paper further reveals the role of green technology innovation and industrial structure upgrading in the process, which provides some theoretical reference for further transformation and green development through technological change to achieve “peak carbon and carbon neutrality.”

## Materials and methods

### Economic strategies

To reveal the role of CETP on carbon emissions, we apply the DID method to assess the two associations following the studies of Yang et al. (2021b), and policy shocks are relatively exogenous to microeconomic agents and do not suffer from reverse causality. The general approach assesses policy effects mainly by setting a dummy variable for the occurrence or non-occurrence of policies and then running a regression. In contrast, the DID model settings are more scientific, can estimate the policy effects more accurately, and avoid the endogeneity problem to a considerable extent. The provincial administrative regions that implemented the optimization and upgrading are defined as the experimental group, while the non-implementing provincial administrative regions were used as the control group. Specifically, this paper uses a dataset with six pilot regions as the experimental group and the remaining 24 regions as the control group. In terms of the pilot period division, because the carbon emission trading system only started in June 2013, 2008–2013 is set as the non-pilot period, and 2014–2020 is set as the pilot period. Moreover, the DID model can largely avoid endogeneity by introducing the dummy variable of CETP implementation or not, which enables us to estimate the effect of CETP implementation more accurately. The estimation model is set as follows:


(1) $$C\,O_{2_{i}{,}_{t}}=\alpha_{0}+\alpha_{1}\,C\,E\,T\,P_{i,t}+\eta_{t}+\mu_{i}+\varepsilon_{i,t}$$

(2) $$C\,O_{2_{i}{,}_{t}}=\alpha_{0}+\alpha \,1\,C\,E\,T\,P_{i,t}+\beta \,C\,o\,n\,t\,r\,o\,l_{i,t}+\eta_{t}+\mu_{i}+\varepsilon_{i,t}$$

Furthermore, to test the carbon emission reduction mechanism of the CETP, the following model is constructed to test the moderating effect.


(3) $$C\,O_{2_{i}{,}_{t}}=\alpha_{0}+\alpha_{1}\,C\,E\,T\,P_{i,t}\times u\,p\,g_{i,t}+\beta \,C\,o\,n\,t\,r\,o\,l_{i,t}+\eta_{t}\,+\mu_{i}+\varepsilon_{i,t}$$

where *CO*_2*i*,*t*_ represents the total carbon emissions in province_  i _ in year *t*. *CETP*_*i*,*t*_ = *tim*e_t_ × d*id*_i_. *time*_*t*_ represents the time grouping variable, 1 for 2014–2020 and 0 for 2008–2013. *did*_*i*_ is the area variable, 1 for pilot areas of carbon emission trading system, and 0 for non-pilot areas. *upg*_*i*,*t*_ is the industrial structure upgrading status in province *i* in year *t*.

*Controli*,*t* are control variables, including population size (*pop*), urbanization rate(*urban*),

human capital status (*edu*), science and technology (S&T) input (*kjtr*), and government intervention(*yszc*). η*t* is a time-fixed effect, μ*i* is an area fixed effect, and ε*i*,*t* is the error term.

### Variable selections

#### Dependent variable

**Carbon emissions (CO_2_).** Various countries officially issue no unified standard for measuring carbon emissions (Wu et al., 2021; Shahzad et al., 2022; Yang et al., 2022c). The default carbon dioxide emission factors of eight types of fossil fuels (Table 1) and the energy fossil fuel consumption of each province are referred to in the 2006 IPCC Guidelines for National Greenhouse Gas Inventories to estimate the carbon emissions of each region in calendar years (Wang et al., 2021; Ren et al., 2022). The specific formula for measuring carbon emissions is as follows:


(4) $$C\,O_{2_{i}{,}_{t}}=k\cdot \sum_{i=1}^{n}E_{i}\cdot \delta_{i}$$

among them, *CO*2 is the carbon emission; *k*(*k* = 44/12) is the carbon dioxide to carbon molecule weight ratio. *Ei* is fossil fuel type *i*. δ*i* is of fossil fuel type*i* emission factor.

**TABLE 1:** Carbon emission factors for each type of fossil fuel.

Fuel types	Default carbon content(kgc/GJ)	Default carbon oxidation rate	Average low level heat generation(KJ/kg,m^3^)	Carbon emission factor(kgc/kg,m^3^)
Coal	25.8	1	20908	0.53943
Coke	29.2	1	28435	0.8303
Crude oil	20	1	41816	0.83632
Gasoline	18.9	1	43070	0.81402
Kerosene	19.6	1	43070	0.84417
Diesel oil	20.2	1	42652	0.86157
Fuel oil	21.2	1	41816	0.88232
Natural gas	15.3	1	38931	0.59564

Based on the above measurement formula and carbon emission coefficients, we calculate the carbon emissions of 30 areas in China from 2008 to 2020. Table 2 reports the carbon emissions of the experimental group, that is, the carbon emissions of the six pilot regions. From Table 2, it can be observed that among the six pilot regions, Beijing, Chongqing, and Hubei have a large decline around 2013, Guangdong has a decline around 2013 but not a large one, Shanghai and Tianjin show an increase around 2013, and the decline effect is shown in 2015.

**TABLE 2:** Carbon emissions in the pilot provinces from 2008 to 2020.

Year	Beijing	Shanghai	Tianjin	Chongqing	Hubei	Guangdong
2008	13.35	25.50	14.02	9.92	27.75	49.10
2009	13.75	27.04	13.75	10.39	27.43	50.32
2010	14.14	27.06	14.87	11.15	29.39	53.68
2011	14.30	29.30	18.88	12.33	32.54	60.18
2012	13.58	29.78	20.74	14.20	36.43	62.85
2013	13.48	29.61	20.51	14.05	36.43	62.38
2014	11.92	31.25	21.18	12.15	32.99	61.96
2015	12.50	28.57	20.25	12.90	33.69	62.28
2016	11.79	29.92	19.88	13.11	33.51	62.76
2017	10.86	30.12	18.59	13.17	33.61	65.46
2018	10.99	30.91	18.70	13.29	34.52	67.77
2019	11.08	28.90	19.09	12.30	34.13	70.84
2020	11.04	30.25	19.18	12.48	36.33	69.55

#### Core explanatory variables

The core explanatory variables in this paper are *did* and *Time*, which are the policy and time dummy variables, respectively. *did* × *Time* indicates the implementation of CETP. The pilot captures whether each province participates in the CETP, and if it is a pilot province, then *did* =  1. Time reflects the implementation time of CETP, and if it is after the implementation of the CETP, then *Time* = 1.

#### Moderating variables

Industrial structure upgrading (*upg*): There are various measures of industrial structure upgrading indicators, such as the ratio of the value-added of secondary and tertiary industries to GDP or the ratio of tertiary industries to the value-added of secondary industries (Qiu et al., 2022). The former is selected in this paper to determine the industrial structure upgrading level of the explanatory variable. Green technology innovation: Green technology innovation is defined as technology innovation that minimizes the total product cost at each stage of the product life cycle innovation process by adhering to the ecological and economic principles and aiming to protect the environment. Because patent applications are more representative of the technological innovation results in the year of application, which often takes one to three years from application to grant (Lin and Ma, 2022), there is uncertainty in patent grants due to many factors such as detection, annual fee payment, and market environment (Sun et al., 2022a). Compared to invention patents, design and utility model patents are at a lower level of technology and easy to learn and imitate, so invention patents are the most representative of a region’s innovation capacity (Behera and Sethi, 2022; Sun et al., 2022b). Therefore, based on the information on green technology innovation activities provided by the IPC, green technology invention patent applications in each region and city were collected on the patent search and analysis system of the State Intellectual Property Office, and the proportion of green technology invention patent applications to the number of patent applications is characterized by following the studies of Lv et al. (2021) and Tang et al. (2021).

#### Control variables

To control the disturbance of the dependent variable by the unknown factors, following Cao et al. (2021); Yang et al. (2022a), and Ren et al. (2022), this paper introduces control parameters, including the region’s population size(*pop*), urbanization rate(*urban*), human capital status (*edu*), S&T input (*kjtr*), and government intervention (*yszc*). Larger population size will be followed by an increase in energy consumption demand, which will help boost carbon emissions. Following Chen et al. (2022), the region’s population size(*pop*) is denoted by the province’s total population at year-end. The urbanization process has contributed to the transformation of human production and lifestyle (Zhu et al., 2021), and the demand for fossil energy has been increasing, affecting carbon emissions. The urbanization rate (*urban*) is calculated as the ratio of the urban population to the total rural population in each province. Human capital can be a constant source of intelligence for carbon-reducing technologies, influencing carbon emissions. Human capital status (*edu*) is calculated as the total number of university students in each province. An increase in investment in S&T can provide financial security for front-end and end-to-end carbon reduction technologies and direct the flow of funds, affecting carbon emissions. S&T input (*kjtr*) is measured by R&D expenditure in each province. The fiscal behavior of local governments directly affects ecosystems and environments. When fiscal behavior is biased toward low-carbon technologies, local fiscal intervention inhibits carbon emissions, and when fiscal behavior is biased toward productive technologies, local fiscal intervention promotes carbon emissions. Local fiscal intervention (*yszc*) is the ratio of local general budget expenditures to regional GDP measured.

### Data

This paper considers the dataset of 30 provincial-level administrative regions from 2008 to 2020 as the subject of investigation in China. All data are derived from the China Statistical Yearbook, the China Macroeconomic Database, and the EPS Global Statistical Data Analysis Platform for the period under investigation. The neighboring value filling method and the mean value filling method are employed to complete the data for the missing data in the statistical sources. Descriptive statistics results of the relevant variable data are detailed in Table 3.

**TABLE 3:** Descriptive statistics.

Variable	Obs	Mean	Std. Dev.	Min	Max
*CO*_*2*_	390	10.240	0.738	8.045	11.928
*CETP*	390	0.108	0.310	0	1
*pop*	390	8.195	0.743	6.317	9.443
*urban*	390	0.570	0.131	0.291	0.896
*edu*	390	12.888	0.800	10.133	14.034
*kjtr*	390	4.069	1.109	1.324	7.064
*yszc*	390	8.165	0.702	5.783	9.766

## Results and discussion

### Discussion of parallel trend test results

**FIGURE 1:**
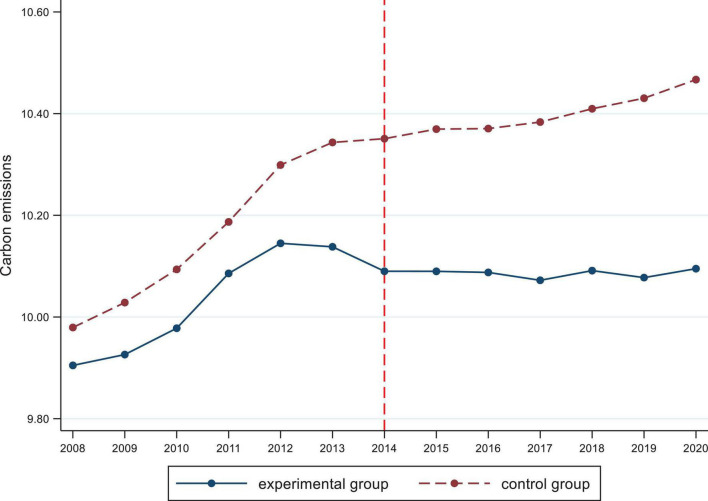
Parallel trend test chart.

An essential assumption for the DID method is that the experimental and control groups have identical trends before CETP implementation (Hao et al., 2022). Specifically, this paper does not show systematic differences in the trends of carbon emissions in the sample provinces before CETP implementation, regardless of whether the CETP implementation is implemented or not. We find that the exogenous shock of the CETP did not influence the carbon emissions trend in the experimental and control groups. Following Tanaka et al. (2015), this paper further portrays whether the experimental group and the control group have consistent trends of change before the pilot carbon emission trading policy by plotting the time trends of the explanatory variables. For the policy node, 2014 is finally chosen as the time node because CETP only started to pass one after another in June 2013. Figure 1 plots the parallel trend after CETP implementation and without CETP implementation, portraying that the trends of carbon emissions before and after CETP implementation are the same as before 2014, implying that the hypothesis of parallel trend in this paper is verified and the double-difference model is applicable.

### Discussion of baseline regression results

This paper investigates the role of carbon emissions levels from CETP implementation using a DID model with the exogenous shock of the CETP implementation. To make the estimation analysis more accurate, the time effects and individual effects in the model are controlled (See Table 2). Column (1) in Table 2 confirms that the regression coefficients of the carbon emission trading pilot policy are positive (P−value < 0.01). Furthermore, the control variables are added for estimation. From column (2), Table 2 reports that the CETP has a significant dampening effect on carbon emission levels, whether the control variables are included or not. Our results are in line with the findings of Wu and Wang (2022) and Yu et al. (2022). Differently from this study, Yu et al. (2022) explore the impact of CETP on carbon emission intensity and a financial performance at the firm level, revealing that CETP can achieve a win–win situation in terms of carbon emission reduction as well as firm performance improvement. Regions with CETP have a more significant carbon emission reduction effect compared to regions that do not implement CETP. From the perspective of the control variables, the process of urbanization in China will generate various energy and carbon emission demands, so it still shows a significant contribution to the regional carbon emission level. However, the effects of regional human capital status (*edu*), science and technology investment(*kjtr*), and local fiscal intervention(*yszc*) on carbon emission reduction in each province and city are not significant at present. Furthermore, in this paper, we estimate the dynamic effects and trends of carbon emissions affected by carbon trading policy (See columns (3) and (4) in Table 4)^4^. By observing the comparison, we find that the regional carbon emission level decreases in the first year after the CETP implementation. In the second to seventh years after the start-up, the carbon emission reduction effect of the CETP is significant, its carbon emission reduction effect coefficients are 0.154, 0.157, 0.186, 0.193, 0.227, and 0.246, respectively, and its carbon emission reduction effect reveals a significant increasing trend.

**TABLE 4:** Baseline estimation results.

Variables	(1)	(2)	(3)	(4)
	
	Average effect		Dynamic effect	
did	−0.186***	−0.163***		
	(0.024)	(0.024)		
did × Year_2014_			−0.135***	−0.143***
			(0.036)	(0.033)
did × Year_2015_			−0.154***	−0.157***
			(0.028)	(0.028)
did × Year_2016_			−0.157***	−0.154***
			(0.031)	(0.032)
did × Year_2017_			−0.186***	−0.168***
			(0.040)	(0.039)
did × Year_2018_			−0.193***	−0.165***
			(0.043)	(0.040)
did × Year_2019_			−0.227***	−0.191***
			(0.048)	(0.046)
did × Year_2020_			−0.246***	−0.199***
			(0.051)	(0.052)
pop		0.718***		0.722***
		(0.224)		(0.229)
urban		1.139***		1.017***
		(0.376)		(0.390)
edu		0.141		0.144
		(0.114)		(0.115)
kjtr		0.002		0.007
		(0.028)		(0.029)
yszc		0.041		0.032
		(0.098)		(0.103)
Time fixed	Yes	Yes	Yes	Yes
Individual fixed	Yes	Yes	Yes	Yes
R-squared	0.980	0.985	0.985	0.985

Robust standard errors in parentheses; *** *p* < 0.01.

On this basis, we add control variables for regression to observe its dynamic effect again. The empirical results show that the effect of CETP on carbon emissions still has the same development trend. Its carbon emission reduction effect will steadily increase as the CETP implementation is postponed. On the one hand, CETP will directly “force” high-emission enterprises to reduce total carbon emissions via technological transformation and other means, thus reducing their relative disadvantage (Yu et al., 2022). The increasing demand for technological innovation will lead to increased knowledge intensity and intensification, accelerated penetration of knowledge-based production factors, and increased labor productivity (Pan et al., 2022). The adjustment of capital and production factors optimizes resource allocation through a low-carbon technology innovation system, thus promoting the rational allocation and utilization efficiency of resources and finally realizing carbon emission reduction. In addition, under the CETP implementation, market selection will eliminate excess capacity and positively affect industrial transformation and upgrading (Wu and Wang, 2022). Industrial restructuring can enhance the linkage between industries and form the basis for promoting the transformation of industrial structure to green and low carbon, thus realizing energy savings for enterprises and promoting regional industrial structure upgrading (Hong et al., 2022).

### Discussion of heterogeneity results

Since there is a heterogeneous distribution of geographical characteristics in the pilot regions where the CETP is implemented, the carbon emission variables also have a strong heterogeneous effect on geographical location. Therefore, this paper divides the thirty provinces in the sample into eastern, central, and western regions based on geographical location and makes interaction terms (dummy variables) between the did variables of whether the CETP is implemented in that year and the eastern, central, and western regions, respectively, to explore the carbon emission reduction effects after the CETP implementation in different regions (see Table 5). Columns (1), (2), and (3) in Table 5 show the impact of carbon emission trading policies on carbon emissions in the eastern, central, and western regions, respectively, confirming that the carbon emission reduction effect of carbon emission trading policy implementation is significant. Its carbon emission reduction effect size is greater in the eastern region (0.149) than in the central region (0.115) than in the western region (0.092).

**TABLE 5:** Heterogeneity results.

Variables	(1)	(2)	(3)
Eastern	−0.149***		
	(0.038)		
Central		−0.115***	
		(0.029)	
Western			−0.092***
			(0.033)
Pop	0.673***	0.484**	0.585***
	(0.221)	(0.216)	(0.222)
Urban	1.306***	2.167***	2.110***
	(0.394)	(0.376)	(0.378)
Edu	0.186	0.144	0.175
	(0.113)	(0.117)	(0.116)
Kjtr	−0.022	−0.015	−0.029
	(0.029)	(0.031)	(0.029)
Yszc	0.030	0.007	−0.012
	(0.103)	(0.102)	(0.101)
Time fixed	Yes	Yes	Yes
Individual fixed	Yes	Yes	Yes
Observations	390	390	390
R-squared	0.984	0.984	0.984

Robust standard errors in parentheses; *** *p* < 0.01 and ** *p* < 0.05.

One potential explanation is that the eastern region occupies an absolute advantage in the number of carbon emission trading pilot areas, so its implementation’s carbon emission reduction effect is more obvious (Hong et al., 2022). In addition, the eastern region has a more developed industrial system, and CETP will, to a certain extent, raise the cost of enterprises with high carbon emission demand, which will directly “force” enterprises to change to a green pattern, thus achieving emission reduction of enterprises and promoting regional industrial structure upgrading (Wu and Wang, 2022). Furthermore, the impact of CETP on high-energy-consuming enterprises is more prominent, directly increasing the operating risks and non-compliance costs, resulting in more emission reduction and innovation drive for such enterprises. Finally, the eastern region has sufficient financial support and talent reserves and is more capable of achieving technological breakthrough innovation and, ultimately, carbon emission reduction against the backdrop of the CETP.

### Discussion of interaction effect results

Based on the above benchmark analysis and heterogeneity test analysis, we concluded that the carbon emission reduction effect of CETP may be greatly influenced by the progress of green technology and industrial structure upgrading. To verify whether our conjecture is correct, the interaction term of green technology innovation and industrial structure upgrading is set in this paper (see Table 6 and Table 7). This paper finds that the interaction term is significantly negative (–0.666), and in the eastern region, its interaction term coefficient is negative (–0.879). Overall, the release of CETP’s carbon emission reduction effect depends on upgrading the industrial structure. Moreover, the coefficient of the interaction term in the eastern region (−0.879) is much higher (Absolute value) than that of the overall sample (−0.666), which is not significant in the central and western regions. It is not hard to understand that sending emission reduction signals by setting the total amount of allowances promotes enterprises to restrain their carbon emission behavior (Tan et al., 2022). This constrained state relies on the low-carbon green transformation of enterprise industrial structure. Carbon emission reduction is a systematic process with systematic requirements for resource allocation, technological innovation, and financial support to ultimately achieve industrial structure optimization and upgrading (Lei et al., 2022). Therefore, this paper argues that CETP’s carbon emission reduction effect depends on upgrading the industrial structure. In addition, there are more pilot areas in the eastern region, and it has a more developed and perfect industrial system, which is conducive to reducing the cost of enterprises with high carbon emission demand, which will directly “force” enterprises to change to a green and low-carbon development pattern, to achieve energy-saving and emission reduction of enterprises (Liu and Sun, 2021). In contrast, the industrial structure upgrading in the central and western regions is limited by the shortcomings of regional capital and technology accumulation, so the carbon emission reduction effect of carbon emission trading is difficult to achieve. This explains why the adjustment effect of industrial structure upgrading in the eastern region is much higher than that of the overall sample.

**TABLE 6:** Industrial structure upgrading interaction results.

Variables	Total	Eastern	Central	Western
CETP*upg	−0.666***			
	(0.221)			
		−0.879***		
		(0.261)		
			0.331	
			(0.302)	
				0.561
				(0.407)
CETP	0.202*	0.409**	−0.265**	−0.385*
	(0.114)	(0.160)	(0.131)	(0.220)
Upg	−0.446*	−0.592**	−0.369	−0.304
	(0.243)	(0.247)	(0.264)	(0.267)
Pop	0.784***	0.671***	0.479**	0.576**
	(0.228)	(0.222)	(0.218)	(0.222)
Urban	0.445	0.957**	2.171***	2.196***
	(0.403)	(0.393)	(0.379)	(0.396)
Edu	0.141	0.151	0.146	0.166
	(0.112)	(0.113)	(0.118)	(0.116)
Kjtr	−0.007	−0.032	−0.020	−0.034
	(0.028)	(0.029)	(0.032)	(0.030)
Yszc	0.019	0.010	−0.021	−0.020
	(0.102)	(0.105)	(0.106)	(0.107)
Time fixed	Yes	Yes	Yes	Yes
Individual fixed	Yes	Yes	Yes	Yes
Observations	390	390	390	390
R-squared	0.985	0.985	0.984	0.984

Robust standard errors in parentheses; *** *p* < 0.01, ** *p* < 0.05, and * *p* < 0.1.

**TABLE 7:** Green technology innovation interaction results.

Variables	Total	Eastern	Central	Western
CETP *gti	0.0003			
	(0.0004)			
		0.0006		
		(0.000343)		
			0.002	
			(0.002)	
				−0.001***
				(0.0003)
CETP	−0.166***	−0.153***	−0.130***	−0.077**
	(0.024)	(0.039)	(0.028)	(0.034)
Gti	−0.0005	−0.0006	−0.0004	−0.0003
	(0.0004)	(0.0004)	(0.0004)	(0.0002)
Pop	0.746***	0.697***	0.520**	0.624***
	(0.230)	(0.225)	(0.223)	(0.229)
Urban	1.161***	1.387***	2.145***	2.121***
	(0.384)	(0.401)	(0.376)	(0.380)
Edu	0.121	0.160	0.133	0.164
	(0.115)	(0.114)	(0.117)	(0.116)
Kjtr	0.005	−0.020	−0.011	−0.025
	(0.028)	(0.029)	(0.031)	(0.029)
Yszc	0.038	0.023	0.003	−0.025
	(0.097)	(0.102)	(0.101)	(0.101)
Time fixed	Yes	Yes	Yes	Yes
Individual fixed	Yes	Yes	Yes	Yes
Observations	390	390	390	390
R-squared	0.985	0.984	0.984	0.984

Robust standard errors in parentheses; *** *p* < 0.01 and ** *p* < 0.05.

As shown in Table 7, the interaction term is positive and insignificant in the national, eastern, and central regions, and the interaction term between CETP and green technology innovation is negative in the western region (*P*-value < 0.01). It indicates that only in the western region, the release of carbon emission reduction role of carbon emission trading policy depends on green technology innovation. In contrast, green technology innovation constrains the carbon emission reduction effect of CETP nationwide and in the eastern and central regions. Due to the economic incentive of CETP, enterprises will pay extra attention to the carbon emission trading market, not only to drive carbon emission reduction by the market mechanism but also to gain additional economic benefits, which causes the “inertia” of enterprises in technological innovation (Zhou and Wang, 2022). At the same time, green technology innovation requires a process, there is a time lag in generating benefits, and the process also requires significant costs, so enterprises tend to choose carbon emission trading compared to the direct economic benefits it can bring. As a result, when companies adopt carbon trading, they allocate a disproportionate share of the carbon reduction effect. The development of green technology innovation will hinder the effect of enterprises in carbon emission reduction. However, it can be found that as green technology innovation tends to mature, it will form a synergistic effect with CETP and jointly promote carbon emission reduction (Dong et al., 2022). In addition, due to the backwardness and geographical location of the western region, the production methods and environmental protection concepts of enterprises are still relatively inadequate, and carbon emissions are relatively high (Cai and Ye, 2022). When the enterprises in the western region adopt carbon emission trading, the application of green technology innovation will quickly complement the backward production technology and produce the effect in time. Meanwhile, the carbon emission reduction generated by green technology innovation may occupy a relatively small proportion of the total starting carbon emissions in the western region. To better achieve the purpose of carbon emission reduction, enterprises will tend to trade more carbon emission rights. With the development of green technology innovation in the western region, CETP’s carbon emission reduction effect will be better (Zhang et al., 2022).

### Discussion of robustness check results

To test the above result’s robustness, the following two steps were used to reveal the full sample. (1) Alternative measures of explanatory variables. In this paper, the regressions are re-validated by replacing the carbon emission variables with SO_2_ emissions (see column (1) in Table 8). (2) All explanatory variables are lagged by one period. Considering the lag of the CETP effect, and to avoid the joint equation bias, the lagged period of carbon emission trading policy implementation and all control variables are taken into the equation for regression (see column (2) in Table 8). Table 8 shows that CETP significantly curbs carbon emissions, which means that the above results are robust.

**TABLE 8:** Robustness check results.

Variables	(1)	(2)
	
	Replacing the dependent variable	All explanatory variables lagged by one period
CETP	−0.257***	−0.142***
	(0.082)	(0.025)
Pop	−0.681	0.593**
	(0.494)	(0.243)
Urban	7.167***	1.236***
	(1.671)	(0.392)
Edu	−0.257	0.105
	(0.272)	(0.119)
Kjtr	−0.183***	−0.006
	(0.066)	(0.029)
Yszc	0.421*	0.126
	(0.224)	(0.095)
Time fixed	Yes	Yes
Individual fixed	Yes	Yes
Observations	390	360
R-squared	0.959	0.986

Robust standard errors in parentheses; *** *p* < 0.01, ** *p* < 0.05, and * *p* < 0.1.

## Conclusion and policy recommendations

The Chinese government incorporated carbon peak and carbon neutrality into its national eco-civilization program to drive economic and social green transitions. Carbon emission trading is an act that attributes carbon emissions to the commodity in which they are produced and permits them to be transacted within the marketplace. This paper adopts a 30-region dataset from 2008 to 2020 in China. CETP’s average, dynamic, and heterogeneous roles are empirically analyzed by constructing the DID model in China. Also, a moderating effect model is constructed to reveal the possible internal mechanisms of the two under industrial structure upgrading and green technology innovation. The paper concludes that CETP has a statistically significant weakening carbon emissions, while its carbon emission reduction effect follows a significant strengthening trend as the policy year of CETP is delayed. CETP significantly inhibits carbon emissions. However, its effect demonstrates a gradual decrease from the eastern to the central and finally to the western regions. CETP can inhibit carbon emissions depending on industrial structure upgrading to a certain extent, and this dependence is significant in the national and eastern regions but not in the central and western regions. The role of CETP on carbon emissions depends on green technology innovation, which is only revealed in the western region and performs as a dampening effect in the national, eastern, and central regions, but not significantly.

The emission reduction effect of CETP is in a desirable state. This paper makes the following recommendations to deal with the double carbon goal and maximize the curbing effect of CETP on carbon emissions. Policymakers should expand the CETP market under the stable operation of the national carbon market, further expand the participating industries in the carbon market, and increase trading varieties. Meanwhile, policymakers should establish a unified standard and normative market not only to refine the national carbon trading market but also to strengthen its effective management and encourage enterprises to achieve carbon emission reduction through carbon exchanges to guide the healthy and effective operation of the national carbon market. Lastly, policymakers should establish a carbon market with Chinese characteristics that combine “market decisions with appropriate government intervention.” For example, for areas where the carbon market is not active, policymakers should play a leading role in the market and use government administrative intervention to improve the quality of carbon trading discretionary.

Policymakers should form a new model of new business types and a new model of online and offline development and promote the high-end development of the industrial structure to optimize the energy mix. In addition, policymakers should also vigorously develop green and clean industries, expand the scope of green technology innovation in the industrial structure from high-energy consumption and high-emission industries to low-emission industries, stimulate the upstream and downstream green demand effects of related industries, guide factors to flow to high-productivity sectors spontaneously, and reduce carbon emissions.

Policymakers should accelerate research on cutting-edge green and low-carbon technologies, vigorously promote high-efficiency and energy-saving technologies, promote the marketization of green technology innovations, and further reduce carbon emissions by developing green and low-carbon technologies. In addition, policymakers should attach importance to the green innovation of enterprises and promote the promotion and application of green technologies. Simultaneously, the government and financial intermediaries provide subsidies and concessions to reduce the cost of innovation investment. Finally, policymakers should strengthen the construction of innovation platforms and form a green technology innovation chain with organic integration of production, education, and research, the effective connection between upstream, middle, and downstream according to local conditions, to reduce carbon emissions.

Although this paper deeply examines the impact of carbon emission trading pilot policy on carbon emissions, some issues still require attention. Due to the limitations in data availability, this paper is unsuccessful in incorporating some key variables, including economic policy uncertainty and institutional environment, into the analysis. Moreover, the effects of the carbon emission trading pilot policy on carbon emissions may be somehow linked in temporal and spatial terms. Therefore, this study can be extended in future by spatial analysis techniques to examine the spatial heterogeneity of carbon emissions from carbon trading pilot policy from the perspective of spatial spillover effects.

## Data availability statement

The original contributions presented in this study are included in the article/supplementary material, further inquiries can be directed to the corresponding author.

## Author contributions

JL: conceptualization, project administration, writing—review and editing, formal analysis, and writing—original draft. WL: data curation, software, visualization, writing—original draft, and writing—review and editing. XZ: methodology, data curation, writing—review and editing, validation, writing—original draft, conceptualization, methodology, funding acquisition, and supervision. All authors contributed to the article and approved the submitted version.

## Footnotes

1
https://www.iea.org/news/global-co2-emissions-rebounded-to-their-highest-level-in-history-in-2021

2 “Visible hand” refers to the government’s efforts to achieve certain goals by intervening, macro-managing or planning and managing to make certain adjustments to economic operations. Public good as a whole has the nature of public goods. By categorizing public service products according to the attributes of competitive, non-competitive, exclusive, and non-exclusive goods, public service products can be classified as private-private goods, private public goods, public private goods, and public-public goods.

3
http://www.gov.cn/xinwen/2022-02/28/content_5676015.htm

4 Dynamic effect test essentially introduces a finite number of time dummy variables and cross-multiplies them with the treatment group dummy variables to examine the significance of the cross-multiplication term. The dynamic effect test examines not only the ex-ante but also the ex-post differences between groups, and if the cross-multiplication term is significant after a certain period (including this period), it indicates that there is some persistent effect of policy implementation.
